# Meditative practices, stress and sleep among students studying complementary and integrative health: a cross-sectional analysis

**DOI:** 10.1186/s12906-022-03582-5

**Published:** 2022-05-05

**Authors:** Adam Sadowski, Ryan S. Wexler, Douglas Hanes, Lita Buttolph, Tediana Torrens, Jillian Moehle, Hadil Sarrar, Joanna Harnett, David T. Zava, Ryan Bradley

**Affiliations:** 1 Helfgott Research Institute, National University of Natural Medicine, Portland, Oregon USA; 2 Faculty of Medicine and Health, School of Pharmacy, The University of Sydney, Sydney, Australia; 3 ZRT Laboratory, Beaverton, Oregon USA; 4 Herbert Wertheim School of Public Health, University of California, La Jolla, San Diego, CA USA

**Keywords:** Medical education, Stress, Sleep, Lifestyle, Naturopathy, Integrative, Cortisol

## Abstract

**Background:**

The International Cohort on Lifestyle Determinants of Health (INCLD Health) is an ongoing, prospective cohort study assessing the health behaviours and lifestyles of higher education students, including their use of specialty diets and complementary and integrative health (CIH) practices. Purpose: This cross-sectional analysis of the INCLD Health cohort aims to (1) evaluate the associations between perceived stress, sleep disturbance, and meditative practices with diurnal salivary free cortisol and (2) evaluate the associations of meditative practices as well as mind-body practices with perceived stress and sleep disturbance.

**Methods:**

Serial multivariable linear regression models, adjusting for sociodemographic and lifestyle behaviours, were used to assess associations of (1) perceived stress, sleep disturbance, and meditative practices with salivary cortisol, and (2) meditative practices as well as mind-body practices with perceived stress and sleep disturbance. Meditative and mind-body practices were evaluated using a stress-management and self-care survey; perceived stress and sleep disturbance were evaluated using the 10-item Perceived Stress Scale (PSS), and the patient reported outcome measures information system-29 (PROMIS-29) sleep sub-score respectively. Salivary cortisol was collected at 4 time points over a 24-hour period and area under the curve (AUC) calculations conducted.

**Results:**

82.5% (*n* = 80) of participants utilized at least monthly meditative practices. Greater disturbed sleep, but not perceived-stress, meditative, nor mind-body practices was independently associated with increased AUC cortisol (b = 0.02, 95% CI: 0.002–0.05, *p* = 0.03) after adjusting for age, sex, race, ethnicity, and BMI. Neither meditative nor mind-body practices were associated with perceived stress or disturbed sleep.

**Conclusions:**

Among INCLD Health participants, greater sleep disturbance, but not perceived stress or meditative practices were associated with daytime cortisol.

**Supplementary Information:**

The online version contains supplementary material available at 10.1186/s12906-022-03582-5.

## Background

Chronic stress, as well as the amount and/or quality of sleep, are associated with greater all-cause mortality [1–5] and are well-established risk factors for cardiovascular disease [1, 2, 6, 7], diabetes [1, 2, 8–10], obesity [1, 2, 11], and hypertension [1, 12]. Additionally, increased stress and inadequate/poor sleep quality are associated with decreased cognitive functioning and academic performance [13–17].

Medical students are exposed to heightened stress over the course of their education [18–20] and their stress levels are greater than age-matched controls [19, 21]. Similarly, a recent meta-analysis of over 14,000 medical students evaluating the association of sleep with academic performance estimated 40% of students experience poor sleep quality and 29% report insufficient sleep [15]. Greater sleep quality and daytime sleepiness but not sleep duration were associated with academic performance, and sleep disruption among medical students was greater than most populations globally. Increased levels of perceived stress and poor sleep, coupled with the academic demands experienced during medical education may each contribute to the high prevalence of burnout amongst medical students [22–24].

In the United States, use of complementary and integrative health (CIH) is highly prevalent with greater than 30% of individuals engaging in some form of CIH, and students receiving higher levels of education routinely demonstrate significantly greater utilization of CIH practices relative to the general population [25–30]. CIH therapies have been utilized to prevent and attenuate perceptions of burnout in medical students and healthcare professionals [31–34]. CIH practices, specifically meditative and/or mind-body practices (e.g., meditation, yoga, Tai Chi) have been increasingly studied to evaluate their effects on stress and sleep with discordant results [33–39]; whereas these practices may be impactful in mitigating perceived stress experienced by medical students [33, 34], few studies have assessed their impacts on improving sleep in this population. Although data exists regarding the use, perception, and efficacy of CIH practices among conventionally trained medical students, a paucity of data exists evaluating the effects of CIH practices on students’ experiences of stress and sleep while receiving an education at an accredited CIH program.

The present study is an ancillary secondary analysis of data collected within the International Cohort on Lifestyle Determinants of Health (INCLD Health) study [40]. This research aimed to (1) evaluate the associations of perceived stress, sleep disturbance, and meditative practices with diurnal cortisol, and (2) identify associations of meditative and mind-body practices with perceived stress and sleep disturbance in students receiving CIH education. An abstract of preliminary data addressing these aims have been published previously [41].

## Material and methods

### Design, setting and participants

This study follows the Strengthening the Reporting of Observational Studies in Epidemiology (STROBE) checklist for cross-sectional studies [42]. The protocol, including data collection instruments and procedures, for the INCLD Health Cohort has been described previously [40]. Briefly, INCLD Health is an ongoing, prospective cohort of English speaking, post-secondary education students, at least 18 years of age, enrolled in accredited CIH education programs from one to six years in length and not in their final year of education (i.e., students in their final year of a four-year program at time of recruitment). Students were included on a rolling basis, had to be willing to complete baseline, 6-month, and yearly follow-up and provide data regarding their demographic information, vital signs, and complete online surveys. Any student in their final year of study, with exception of single-year programs. Were excluded from participation. Participants were recruited through convenience sampling utilizing campus-wide flyers, announcements, e-mails, social media, and the flagship university’s (National University of Natural Medicine, Portland, Oregon, USA) website. Initially study visits were conducted in-person at National University of Natural Medicine’s Helfgott Research Institute, however, due to the ongoing COVID-19 pandemic, study visits were conducted via Zoom. Incentives for participation included comprehensive laboratory, nutritional, and fecal analyses in addition to a reusable drinking mug at no cost to the participants.

In this cross-sectional, secondary analysis of the INCLD Health Cohort study, students who completed their initial study intake between October 2019 and February 2021 were included for analysis. This work was supported by the Helfgott Research Institute of the National University of Natural Medicine (NUNM) and has been approved by the NUNM Institutional Review Board (approval no.: RB091218.)

### Outcomes

The primary outcomes of interest were the associations of perceived stress and sleep disturbance as well as the use of meditative practices with physiologic measures of salivary free cortisol. Both perceived and physiologic stress were assessed, and to our knowledge, no study to date has evaluated the association of the validated instrument, 10-item Perceived Stress Scale (PSS-10) with measures of salivary cortisol levels [43]. Secondary outcomes of interest were the associations of meditative practices (operationalized as use of one or more of the following: Vipassana, Zen Buddhist meditation, Mindfulness-Based Stress Reduction, Mindfulness-Based Cognitive Therapy or prayer) and mind-body practices (operationalized as use of one or more of the following: Tai Chi, Qigong, yoga) with perceived stress and sleep disturbance.

### Data collection

At the initial 90-minute baseline visit, following signed informed consent, participants provided self-reported anthropometric measurements (height, weight, waist circumference, blood pressure, and heart rate), responses to several surveys and questionnaires utilizing REDCap® software, a validated online food frequency questionnaire software (Vioscreen™ by VioCare, Princeton, NJ, USA) and reviewing instructions for completion of an optional at-home salivary hormone testing (ZRT Laboratories, Beaverton, OR, USA). Key aspects of questionnaires included sociodemographic data, use of medications, dietary and herbal supplements, legal and illicit substance use, as well as past and current physical and mental health-related comorbidities (see Additional file 1). Participants were reminded via email 1-week following their visit to complete surveys or provide missing survey data. Participants were reminded via email 1-day prior to their scheduled sample collection date to collect their salivary cortisol specimens and return their collection kits to a central location at the university’s research center (Table 1 here).

**Table 1:** Baseline Characteristics of cohort participants with AUC Cortisol data

Characteristic	*n* = 98
Age, median (IQR), years	28 (25.0–32.0)
Female Sex, number (%)	79 (84.0%)
Race/Ethnicity, n(%)	
White, Non-Hispanic	73 (74.5%)
Black, Non-Hispanic	2 (2.0%)
Latino/Latina/LatinX	9 (9.2%)
Asian	5 (5.1%)
Middle Eastern	2 (2.0%)
Other	7 (7.1%)
BMI, mean (SD)	23.81 (4.11)
Current Smoker, n(%)	12 (12.24%)
Alcohol Consumption, n(%)	*n* = 98
Never	21 (21.4%)
1x/month	8 (8.2%)
2-3x/month	33 (33.6%)
1x/week	5 (5.1%)
2x/week	15 (15.3%)
3-4x/week	11 (11.2%)
5-6x/week	3 (3.1%)
1x/day	1 (1.0%)
>2x/day	1 (1.0%)
Physical Activity Level, n (%)	
Sedentary	9 (9.4%)
Low Activity	26 (27.1%)
Active	44 (45.8%)
Very Active	14 (14.6%)
Extremely Active	3 (3.1%)
AUC Cortisol (ng/mL), median (IQR)	
Morning	6.1 (4.3–7.9)
Noon	1.9 (1.5–2.7)
Evening	1.2 (0.9–1.7)
Night	0.65 (0.5–1.0)
PSS-10 score, mean (SD)	15.28(6.16)
PROMIS-Sleep, mean (SD)	48.25(7.44)
Meditative Practice, n(%)	*n* = 80
Never	17(17.5%)
1x/month	9 (9.3%)
2-3x/month	11 (11.3%)
1x/week	15 (15.5%)
2-3x/week	13 (13.4%)
>3x/week	32 (33%)
Mind Body Practices, n(%)	*n* = 70
Prayer ≥ 1 per week, n(%)	32 (32.9%)
Asana Yoga ≥ 1 per week, n(%)	28 (28.6%)
Pranayama Yoga ≥ 1 per week, n(%)	17 (17.3%)
Tai-Chi ≥ 1 per week, n(%)	18 (18.5%)
Other ≥ 1 per week, n(%)	86 (88.7%)

*IQR* Interquartile range, *BMI* Body Mass Index, *AUC* Area Under the Curve, *PSS-10* 10-item Perceived Stress Scale, *PROMIS-Sleep* Patient Reported Outcome Measures Information System-Sleep Score, *ng/mL* nanogram per milliliter

### Measures

#### Sociodemographics

Sociodemographic data included: age (years), sex (male or female), race (White or Caucasian, Black or African American, Asian, Middle Eastern, Native Hawaiian or other Pacific Islander, American Indian or Alaskan Native, multiple races, or unknown/other), ethnicity (Non-Hispanic or Latino/Latina/LatinX, Hispanic or Latino/Latina/LatinX, or unknown); smoking status (ranging from never to at least twice per day over the past month); and standardized alcoholic beverage consumption (categorized identically to smoking status) were collected. Due to the low frequency of representation of individual non-White races and ethnicities (*n* = 25 total), race and ethnicity were collapsed into two categories: White, non-Hispanic or Other. Smoking status was dichotomized as smokers and non-smokers whereas alcohol consumption was categorized as consuming ≥1 standardized alcoholic beverage per week or < 1 per week for analysis. Participants were otherwise able to select from the following responses for alcohol consumption: (1) Never (2) one time in the last month (3) 2–3 times in the last month (4) once per week (5) twice per week (6) 3–4 times per week (7) 5–6 times per week (8) once per day (9) two or more times per day.

#### Perceived stress

The 10-item Perceived Stress Scale (PSS-10) was utilized to measure participants’ perceived levels of stress in their lives over the past month [43]. For example, “In the last month, how often have you felt nervous and stressed?” Responses to each item of the PSS-10 range from 0 (never) to 4 (very often) for a maximum total score of 40, where higher scores indicate greater levels of perceived stress. Additional questions ask about feelings of being upset, inability to control important things, confidence in the ability to handle personal problems, ability to cope with responsibilities, organization, and ability to complete tasks (See Additional file 2). The PSS has consistently shown to have a Cronbach’s alpha greater than 0.70 (range 0.74–0.91) and a test-retest reliability above 0.70 [43].

#### PROMIS-29 sleep disturbance

Perceived sleep-disturbance was assessed with the sleep subscale component of the validated, patient reported outcome measures information system 29 (PROMIS-29) [44]. The PROMIS-29 sleep subscale consists of four items assessing sleep quality, ability to fall asleep, and ability to maintain sleep over the previous 7 days. Raw scores are then transformed to a T-score, with larger T-scores representing greater sleep disturbance (see Additional file 3) [44]. PROMIS-29 has demonstrated high internal consistency amongst a variety of patient populations with Cronbach’s 𝛼 > 0.75 [44–48], a test-retest reliability (*r* = 0.831; ICC = 0.70–0.82) [47–49], and a strong correlation with the Pittsburgh Sleep Quality Index (*r* = 0.85) [49].

#### Physical activity

Self-reported activity levels and body mass index (BMI) were obtained through a comprehensive online food frequency questionnaire software (Vioscreen™)^.^ Activity levels were categorized as sedentary; low activity (30–60 minutes of daily moderate physical activity); active (at least 60 minutes of daily moderate physical activity); very active (at least 60 minutes of daily moderate physical activity plus an additional 60 minutes of vigorous activity or 120 minutes of daily moderate activity); and extremely active (daily intense activity or activity beyond those defined in very active).

#### Stress management and self-care

An eight-question survey, modified from the 2012 National Health Interview Survey (NHIS) complementary and alternative health survey, was used to identify the type and frequency of self-care practices performed by study participants over the past month [50]. Self-care practices consisted of meditative practices (at least one of the following: Vipassana, Zen Buddhist meditation, Mindfulness-Based Stress Reduction, or Mindfulness-Based Cognitive Therapy), and mind-body practices (at least one of the following: prayer, asana yoga, pranayama yoga, Tai-Chi, Qigong, or other self-care activities). For all self-care practices, participants were able to choose from the following responses: (1) Never (2) once in the last month (3) 2–3 times in the last month (4) once per week (5) 2–3 times per week (6) more than three times per week.

#### Cortisol

Salivary cortisol has demonstrated a sensitivity and specificity greater than 90% in states of hypercortisolism and was collected by participants per instructions for a commercially available clinical laboratory test (ZRT Laboratory, Beaverton, OR) after providing informed consent for laboratory testing [51]. Participants who consented to laboratory testing were mailed a testing kit by the study coordinator to their provided address of residence. Returned samples were inspected by the study coordinator for completeness and frozen until sent to ZRT laboratories on a quarterly basis for sample processing. Saliva samples were collected at four time points throughout a 24-hour period within three-months after their baseline visit. Participants were instructed to collect cortisol samples at general time points as opposed to a strict time frame to ensure an adequate number of samples could be collected for analysis. Participants were encouraged to collect all morning samples within 30 minutes of waking, around times related to meals (i.e., before lunch and dinner), and before bed, on a single day.

### Statistical analysis

**Fig. 1:**
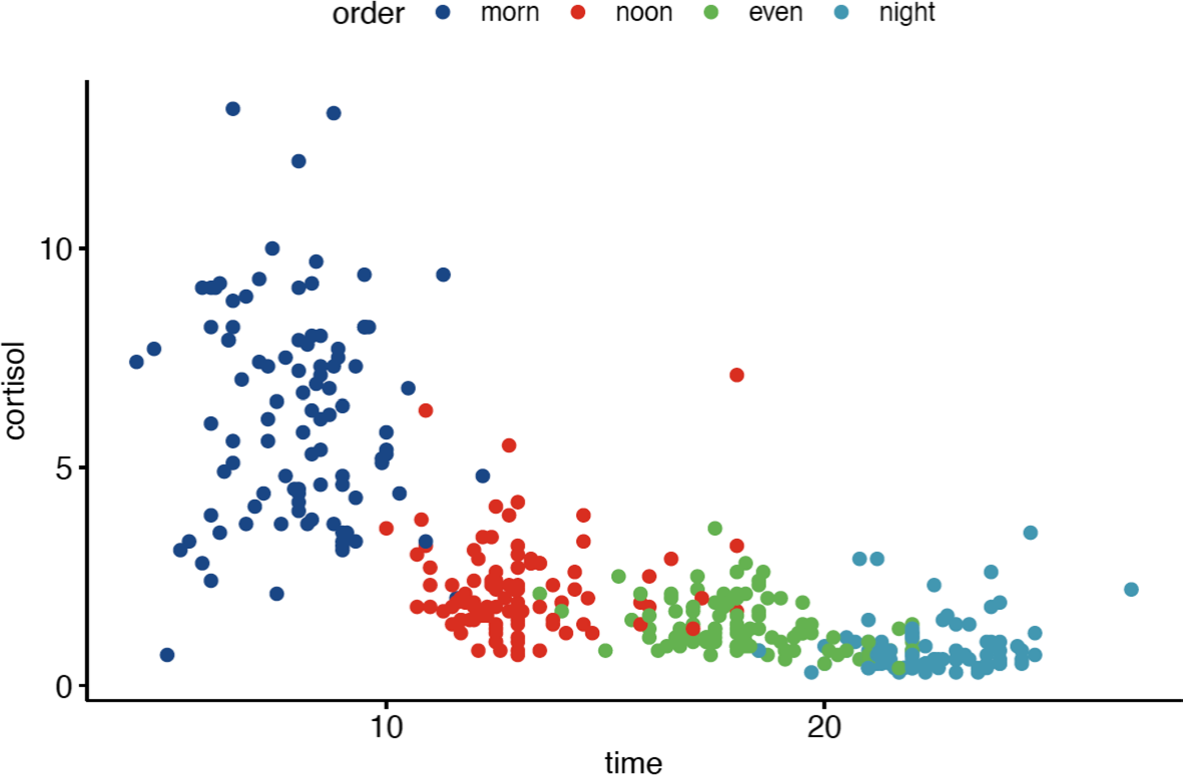
Scatter plot of individual participant data over 24-hours. Time axis is in number of hours past midnight. Cortisol units is ng/mL. The participant with outlying cortisol measures can be seen with noon cortisol measured at > 18.00 hours and with value greater than 5 ng/mL. Morn (morning cortisol collection), noon (afternoon cortisol collection time), even (evening cortisol collection), night (night-time cortisol collection)

All data were analyzed with R software® version 4.0.2 (Vienna, Austria), using a significance level of *p* < 0.05 [52]. Power calculations (made using the R package pwr) showed that, with 94 participants and a standard alpha of 0.05, we would have 84% power to detect a simple correlation of *r* = 0.3 and 81% power to detect a significant regression coefficient in the fully adjusted model with a standardized beta of 0.1, both of which are consistent with a *medium* sized effect, according to the benchmarks developed by Cohen [53]. Frequencies and percentages are reported for categorical variables and means (±SD) for continuous variables, unless otherwise specified. The current paper uses only participants in the INCLD cohort with cortisol measures. Partially missing cortisol data from three participants were imputed using linear interpolations. To account for variations in salivary cortisol specimen collection times, cortisol results were transformed into a time-weighted daytime average area under the curve (AUC). One participant with extreme cortisol data was excluded from the primary analysis (Fig. 1). Participants with missing data from questionnaires and surveys, despite attempts to contact participants to complete surveys and questionnaires, were excluded from analyses using those measures. Additionally, the category of “other” within self-care practices were removed as they were largely not representative of either meditative or mind-body practices (i.e., hiking). Daytime cortisol was summarized as a time-weighted AUC calculation, using four individual measurements, according to the formula:

$$auc=\frac{m. cort+2\ast n. cort+2\ast e. cort+n. cort}{6\ast \left(n. time-m. time\right)}$$

For the sake of regression analysis, participants were categorized as *meditators* if they endorsed engaging in any one of the following meditation practices: Vipassana, Zen Buddhist meditation, Mindfulness-Based Stress Reduction, Mindfulness-Based Cognitive Therapy, or prayer. Participants who did not engage in any one of the practices were categorized as *non-meditators.* Participants who engaged in any one of the following mind-body practices: Tai Chi, Qigong, or yoga (asana or pranayama) were categorized as *users of mind-body practices*. Participants who did not engage in any of the mind-body practices were categorized as *non-users of mind-body practices*.

Unadjusted linear regression models were performed to identify crude associations of PSS-10 and PROMIS-29 sleep scores as well as the use of meditative practices with AUC cortisol. The crude associations were then adjusted using stepwise addition of covariates adjusted for age, sex, and ethnicity with age as a continuous variable (years) and sex, race and ethnicity dichotomized as White, non-Hispanic or other. The model was further adjusted for BMI as a continuous variable (kg/m^2^); and adjusted for alcohol consumption, smoking status, and physical activity level. Due to low counts, extremely active and very active physical activity levels were combined as one category. Given the evidence supporting a relationship of poor sleep and heightened stress with dysregulated glucose control, the associations of perceived stress and disturbed sleep with AUC cortisol was fully adjusted with glycated hemoglobin, added as a continuous variable (percentage of glycosylated hemoglobin) for the associations. Results following the incorporation of glycated hemoglobin should be interpreted as exploratory [1, 2, 54–56].

The associations of meditative practices and mind-body practices with PSS-10 and PROMIS-29 sleep scores were examined using the same stepwise addition of covariates to serial models as described above.

## Results

### Study population

Data was available for (*n* = 98) participants who completed salivary cortisol collections during at least two different times within 24-hours. The sample characteristics are shown in Table 1. Sociodemographic characteristics of the sample were as follows: median age of participants was 28.0 years (IQR 25.0–32.0) and participants predominantly were female (84.0%), White Non-Hispanic (74.5%), non-smokers (87.8%), drank less than one alcoholic beverage per week (63.3%), and engaged in some form of meditative practice (82.5%). Less than 10% of participants self-reported sedentary activity and average PSS-10 and PROMIS-29 sleep scores were 15.28 (6.16) and 48.25(7.44), respectively. All 98 participants completed PSS-10 and PROMIS-29 sleep questionnaires, with 97 completing surveys assessing meditative and mind-body practices. Individual participant cortisol data, prior to taking the time-weighted daytime average can be seen in Fig. 1.

### Associations of perceived stress, sleep disturbance, and meditative practice with AUC cortisol

PSS-10 and use of meditative practices were not significantly associated with AUC cortisol in crude or serially adjusted multivariable linear regression analyses (Tables 2, 3, 4), however, disrupted sleep was associated with AUC cortisol when unadjusted (b = 0.02, 95% CI: 0.02–0.04, *p* = .03) and after adjusting for age, sex, race and ethnicity (: b = 0.02, 95% CI: 0.003–0.04, *p* = .02), and BMI (b = 0.02, 95% CI: 0.002–0.05, *p* = .03). Associations approached significance after further adjusting for smoking status, alcohol consumption, and physical activity level (b = 0.02, 95% CI: − 0.0003-0.05, *p* = 0.053).

**Table 2:** Associations of Perceived Stress with time-weighted AUC Cortisol

Perceived Stress			
AUC Cortisol	b	95% CI	*P* value
Unadjusted	0.01	−0.01 – 0.03	0.47
Age, Sex, Race/Ethnicity	0.01	−0.01 – 0.04	0.35
BMI	0.01	−0.01 – 0.04	0.40
Alcohol, Smoking, Physical Activity	0.01	−0.01 – 0.04	0.36
Fully Adjusted	0.01	−0.02 – 0.04	0.51

Table shows regression coefficient with 95% Confidence interval (95% CI) and *p*-value for effect of PSS-10 (10-item Perceived Stress Scale) on AUC (Area Under the Curve) cortisol, in each model. Crude model (Unadjusted); adjusted for age, sex, race/ethnicity; Body mass index (BMI); Alcohol consumption, use of tobacco, and physical activity level; Fully adjusted model includes the addition of hemoglobin A1c (Fully Adjusted); b (beta-coefficient)

**Table 3:** Associations of meditative practices with time-weighted AUC Cortisol

Meditative Practice			
AUC Cortisol	b	95% CI	*P* value
Unadjusted	−0.03	− 0.39 – 0.34	0.88
Age, Sex, Race/Ethnicity	0.02	−0.4 – 0.42	0.9
BMI	0.05	−0.4 – 0.5	0.8
Alcohol, Smoking, Physical Activity	0.11	−0.3 – 0.53	0.6

Table shows regression coefficient with 95% Confidence interval (95% CI) and *p*-value for effect of PSS-10 (10-item Perceived Stress Scale) on AUC (Area Under the Curve) cortisol, in each model. Crude model (Unadjusted); adjusted for age, sex, race/ethnicity; Body mass index (BMI); Alcohol consumption, use of tobacco, and physical activity level; Fully adjusted model includes the addition of hemoglobin A1c (Fully Adjusted); b (beta-coefficient) *p < 0.05

**Table 4:** Associations of disturbed sleep with time-weighted AUC Cortisol

Disturbed Sleep			
AUC Cortisol	b	95% CI	*P* value
Unadjusted	0.02	0.02–0.04	0.03^*^
Age, Sex, Race/Ethnicity	0.02	0.003–0.04	0.02^*^
BMI	0.02	0.002–0.05	0.03^*^
Alcohol, Smoking, Physical Activity	0.02	−0.0003 – 0.05	0.053
Fully Adjusted	0.01	−0.01 – 0.04	0.2

Table shows regression coefficient with 95% Confidence interval (95% CI) and *p*-value for effect of PSS-10 (10-item Perceived Stress Scale) on AUC (Area Under the Curve) cortisol, in each model. Crude model (Unadjusted); adjusted for age, sex, race/ethnicity; Body mass index (BMI); Alcohol consumption, use of tobacco, and physical activity level; b (beta-coefficient)

### Associations of meditative and mind-body practices with perceived stress and sleep disturbance

There were no statistically significant associations of meditative or mind-body practices with PSS-10 or PROMIS-29 sleep scores (Tables 5 and 6) in crude or serially adjusted multivariable linear regression analyses.

**Table 5:** Associations of meditative practices with perceived stress and disturbed sleep

Meditative Practice			
**PSS-10**	b	95% CI	*P* value
Unadjusted	−2.0	−5.3 – 1.2	0.22
Age, Sex, Race/Ethnicity	−1.9	0.005–5.0	0.3
BMI	−1.2	−4.8 – 2.5	0.53
Alcohol, Smoking, Physical Activity	−0.73	−4.3 – 2.9	0.7
Fully Adjusted	−1.1	−5.0 – 2.9	0.6
**PROMIS-Sleep**	b	95% CI	*P* value
Unadjusted	0.25	−3.7 – 4.22	0.9
Age, Sex, Race/Ethnicity	0.25	−3.9 – 4.4	0.9
BMI	1.4	−2.8 – 5.5	0.52
Alcohol, Smoking, Physical Activity	2.4	−1.6 – 6.4	0.23
Fully Adjusted	3.0	−1.2 – 7.3	0.2

Table shows regression coefficient with 95% Confidence Interval (95% CI) and *p*-value for effect of meditative practices on PSS-10 (10-item Perceived Stress Scale) and PROMIS-29 (Patient Reported Outcome Measures Information System-29 Sleep Score), in each model. Crude model (Unadjusted); adjusted for age, sex, race/ethnicity; Body mass index (BMI); Alcohol consumption, use of tobacco, and physical activity level; Fully adjusted model includes the addition of hemoglobin A1c (Fully Adjusted); b (beta-coefficient)

**Table 6:** Associations of disturbed sleep with time-weighted AUC Cortisol

Mind-body Practices			
**PSS-10**	b	95% CI	*P* value
Unadjusted	−0.08	−2.8 – 2.9	0.96
Age, Sex, Race/Ethnicity	0.30	0.07–25.9	0.84
BMI	0.93	−2.1 – 4.0	0.54
Alcohol, Smoking, Physical Activity	0.65	−2.3 – 3.6	0.66
Fully Adjusted	0.87	−2.4 – 4.2	0.6
**PROMIS-Sleep**	b	95% CI	*P* value
Unadjusted	−0.6	−2.7 – 3.9	0.72
Age, Sex, Race/Ethnicity	−0.7	−4.2 – 2.8	0.70
BMI	0.1	−3.4 – 3.6	0.96
Alcohol, Smoking, Physical Activity	−0.2	−3.5 – 3.1	0.9
Fully Adjusted	0.12	−3.5 – 3.7	0.95

Table shows regression coefficient with 95% Confidence Interval (95% CI) and *p*-value for effect of mind-body practices on PSS-10 (10-item Perceived Stress Scale) and PROMIS-29 (Patient Reported Outcome Measures Information System-29 Sleep Score), in each model. Crude model (Unadjusted); adjusted for age, sex, race/ethnicity; Body mass index (BMI); Alcohol consumption, use of tobacco, and physical activity level; Fully adjusted model includes the addition of hemoglobin A1c (Fully Adjusted); b (beta-coefficient)

## Discussion

In this secondary analysis of data obtained during an ongoing longitudinal study, the INCLD Health Cohort, sleep disturbance as measured by the PROMIS-29 sleep sub score, was associated with an increase in time-weighted AUC cortisol independent of age, sex, race, ethnicity, and BMI. Although the effect did not retain significance after adjusting for smoking status, alcohol consumption, and physical activity this may partly be explained by overfitting of the regression analysis as the number of predictor degrees of freedom doubled when adjusting for BMI to adjusting for alcohol, smoking, and physical activity.

The positive association of disrupted sleep with AUC cortisol in this sample, although small, is consistent with the overall literature supporting the well-established biological relationship between sleep and cortisol. (57–59) Although individuals may not perceive a stressor(s) as stressful, physiologic adaptations, such as increased cortisol output secondary to disrupted sleep, influence the neuroendocrine function via the hypothalamic pituitary-adrenal axis [57, 58]. Also, the physiologic effect of cortisol to induce insulin resistance, and therefore impact glucose metabolism, is well documented [59] and may act as a mediator in the poor health outcomes associated with disturbed sleep, including metabolic syndrome and type 2 diabetes [60]. Thus, salivary cortisol testing in patients reporting sleep disturbance may be warranted in future research. However, while multiple interventions exist for addressing sleep disturbance specifically (i.e., cognitive behavioral therapy for insomnia, benzodiazepines, benzodiazepine receptor agonists, melatonin, etc.) their ability to affect glucose metabolism is inconclusive, and solely focusing on improvements in sleep may not be adequate [61]. Given that mind-body practices have been shown to improve sleep [36] *and* reduce cortisol [62] they may be potential therapeutic options for improving health outcomes associated with disrupted sleep, however, further well conducted randomized controlled trials utilizing active controls are needed. Of note, although we considered sleep quality an independent variable, and cortisol the dependent variable, the actual mechanistic relationship between the two is well established as bi-directional, thus the true directionality in this current cohort unknown [63–66]. Additionally, identifying which specific mind-body practice(s) that provide the greatest magnitude of effect can help guide recommendations provided to both students and the general population.

Interestingly, there were no associations of perceived stress or meditative practices with time-weighted AUC cortisol. The lack of an association between perceived stress with salivary cortisol, is not isolated to our study [67–69] and can be partly explained by temporal asynchrony (i.e., the retrospective nature of perceived psychological stress vs. in the moment physiological cortisol assessment), differences in fundamentals of stress measurement (e.g., capturing cognitive, behavioral, or emotional stress, etc.) and confounding from psychological traits biasing self-reported measurements [70–73]. A review by Campbell and Ehlert was only able to identify approximately 25% of studies included in their review as demonstrating an association between perceived and physiological stress, when assessed prior to, during, and after experiencing the Trier Social Stress Test; further supporting the notion of temporal asynchrony, whereby rapid emotional states occurred prior to appropriate cortisol output responses [73]. In our cohort, the lack of association of perceived stress with physiological stress may also be in part due to most participants entering the study relatively early on in their education (e.g., first year students) or at the start of a new term when their demands are at a nadir. Whether this is possibly due to associations between different stress measures having weaker associations at low levels of stress, is unclear and highlights a gap in research warranting further evaluation.

The lack of an association of meditative practices with time-weighted AUC cortisol contrast with Sanada et al.’s meta-analysis of experimental data which found a low to moderate effect size in improving salivary cortisol measures following mindfulness-based interventions in healthy adults [62]. However, another review suggested mind-body interventions provided inconsistent effects on salivary cortisol [74]. If meditative practices influence cortisol, the high frequency of meditative practices in this sample may have narrowed the range of cortisol concentrations measured, and thus reduced possible associations present in other populations or those achievable when introducing meditative practices to non-meditators.

In this study, there was a lack of an association of meditative or mind-body practices with perceived stress, or sleep disturbance despite most participants engaging in some form of meditative and/or mind-body practice at least once per week. These findings are also in contrast to Spinelli et al. demonstrating low-to-moderate effect sizes for mindfulness strategies to improve stress perceptions amongst medical trainees as well as healthcare providers, when newly introduced to these practices [34]. Another review suggested similar effect sizes to that of Sanada et al. when meta-analyzing the effects of mindfulness meditation on sleep quality [36]. The cross-sectional nature of the present analyses, i.e., the absence of data prior to the onset of meditative practices in this sample, preclude drawing conclusions about potential moderating effects of these practices in the current sample. Training effects/adaptation to the practices are also potential explanations for the lack of association here.

Strengths of this study include the unique cohort of individuals – post secondary education students studying CIH practices. This subpopulation of medical and health education students is unique in their diverse and high utilization of CIH, providing a novel opportunity to study the effects of health and lifestyle practices in a young adult population amongst those receiving an evidence based CIH education. Furthermore, 97% of the participants were able to provide adequate samples for all four salivary cortisol measures, and thus interpolation of missing data is unlikely to have significantly impacted results. The use of both subjective and objective measures of stress are additional strengths, although we did not measure internal reliability within the INCLD cohort. This is also the first study to our knowledge that directly assesses the relationship of the PSS-10 questionnaire with physiologic diurnal salivary cortisol levels.

Our findings are limited to the cross-sectional analysis of baseline data, as well as the relatively small sample size. However, our sample size is approximately 25% of the *eligible* student population. Additionally, we intend to publish data on longitudinal changes in perceived stress, sleep, and cortisol, as well as changes in meditative and mind-body practices from the initial study visit as participants progress through their education, which should provide new insights on the directionality of the sleep/cortisol associations observed here. Due to a lower than anticipated number of students enrolled in the cohort, our analysis includes students receiving an education during the COVID-19 pandemic. How the effects of lockdown restrictions, social isolation, online curriculums, and the physical and mental health impacts associated with the pandemic confound the results in this cohort are unknown. However, measures of cortisol, sleep, stress, and all demographic characteristics, except for age and frequency of at least weekly Tai-Chi, showed no discernible differences between participants who enrolled prior to or after the beginning of the pandemic (Table 2 Additional file 4). Additionally, it is unknown how generalizable our findings may be as our sample consists primarily of young, healthy, physically active, females receiving higher education with a high prevalence of meditative or mind-body practices.

## Conclusion

In this cross-sectional analysis of data obtained during the initial study visit of the INCLD Health cohort, disturbed sleep but not perceived stress or meditative practices are associated with diurnal AUC cortisol, with an attenuation in the relationship after adjusting for smoking status, alcohol consumption, and physical activity level. Neither meditative practices nor mind-body practices were associated with perceived stress or disturbed sleep and additional studies are needed to understand the relationship between perceived stress and diurnal cortisol response, specifically applying the PSS-10 scale.

## Supplementary Information

**Additional file 1.**

**Additional file 2.**

**Additional file 3.**

**Additional file 4.**

## Data Availability

The dataset supporting the conclusions of this article can be made available from the corresponding author on reasonable request.

## Abbreviations

CIH: Complementary and Integrative Health
INCLD: International Cohort on Lifestyle Determinants of Health
STROBE: Strengthening the Reporting of Observational Studies in Epidemiology
NUNM: National University of Natural Medicine
PSS-10: 10-item Perceived Stress Scale
PROMIS-29: Patient Reported Outcome Measures Information System 29
AUC: Area Under the Curve
