# Supplementary material for: Meditative practices, stress and sleep among students studying complementary and integrative health: a cross-sectional analysis
Source: BMC Complement Med Ther. 2022 May 5;22:127. doi: 10.1186/s12906-022-03582-5 (PMC9070612; doi:10.1186/s12906-022-03582-5)
Supplement: Supplementary file 1 — Additional file 1. [file 12906_2022_3582_MOESM1_ESM.pdf]

Demographics

All information obtained through this questionnaire will be kept confidential. Your responses will become part of your research for this study but will not become part of your medical records at NUNM.

Please complete the survey below.

Thank you!

First name ____________________

Middle name ____________________

Last name ____________________

Daytime phone number: ____________________

Evening phone number: ____________________

Email address: ____________________

Address: ____________________

	Yes	No
May we send mail to this address?	☐	☐

Date of birth: ____________________

Current age: ____________________

Sex Assigned at Birth
- ◯ Male
- ◯ Female
- ◯ Intersex

Gender Identity:
- ◯ Cis-male
- ◯ Trans-male
- ◯ Cis-female
- ◯ Trans-female
- ◯ Non-binary
- ◯ Prefer not to say

Sexual Partners	☐ Cis-female<br>☐ Trans-female<br>☐ Cis-male<br>☐ Trans-male<br>☐ Intersex<br>☐ Non-binary<br>☐ Prefer not to say<br>☐ Other<br>(Please check all that apply)
Ethnicity:	◯ Hispanic or Latino/Latina/LatinX<br>◯ Not Hispanic or Latino/Latina/LatinX<br>◯ Unknown/Not Reported
Race:	◯ Black or African American<br>◯ Asian<br>◯ Middle Eastern<br>◯ Native Hawaiian or other Pacific Islander<br>◯ American Indian/Alaska Native<br>◯ White/Causasian<br>◯ More than one race<br>◯ Other or unknown
School Information:<br>Program (select all that apply)	☐ Naturopathic Medicine<br>☐ Classical Chinese Medicine<br>☐ Nutrition<br>☐ Integrative Medicine Research<br>☐ Integrative Mental Health<br>☐ Global Health<br>☐ Ayurveda<br>☐ Undergraduate Programs<br>(Please check all that apply)
Year in school:	◯ Year 1<br>◯ Year 2<br>◯ Year 3<br>◯ Year 4<br>◯ Year 5<br>◯ Year 6<br>◯ Year 7<br>◯ Year 8 or above
Previous degree(s):	☐ BA/BS<br>☐ MA/MS<br>☐ MD or DO<br>☐ DC<br>☐ ND<br>☐ MPH<br>☐ PhD<br>☐ Other<br>(Please check all that apply)
If Other, please specify:	________________________________

Confidential

Vitals and Anthropometrics

Record ID:	______________________
Study Visit Date:	______________________
Systolic Blood Pressure (mmHg) - while seated	______________________
Diastolic Blood Pressure (mmHg) - while seated	______________________
Pulse (beats/min)	______________________
Height (in)	______________________
Weight (lbs)	______________________
Waist Circumference	______________________
Reproductive Status:	◯ Menstruating (no hormone supplements)<br>◯ Hysterectomy (ovaries removed)<br>◯ Menopausal<br>◯ Post-menopausal<br>◯ Hormone replacement<br>◯ Hormonal contraception<br>◯ Pregnant<br>◯ Not applicable
If so, what was the date of first day of your most recent menstrual cycle?	______________________<br>((mm/dd/yyyy))
Number of Medications	______________________
Number of Supplements	______________________

Health History Questionnaire

All information obtained through this questionnaire will be kept confidential. Your responses will become part of your research record for this study but will not become part of your medical records at NUNM.

Medical History

Please indicate whether you have never had, had in the past, or currently have any of the following conditions. Please mark 'past' or 'current' for ONLY those conditions which you have received a formal diagnosis by a physician or other healthcare provider.

Cardiovascular System

	Never	Past	Current
Heart Disease	○	○	○
High Cholesterol	○	○	○
High Blood Pressuure	○	○	○
Anemia	○	○	○
Thrombophelbitis	○	○	○

Respiratory System

	Never	Past	Current
Asthma	○	○	○
Chronic bronchitis	○	○	○
Emphysema	○	○	○
Pneumonia	○	○	○
Chronic or regular common colds	○	○	○
Tuberculosis	○	○	○
Obstructive sleep apnea	○	○	○

Dermatologic

	Never	Past	Current
Contact dermatitis	○	○	○
Eczema	○	○	○
Psoriasis	○	○	○
Acne	○	○	○

Gastrointestinal

	Never	Past	Current
Hepatitis A	○	○	○
Hepatitis B	○	○	○
Hepatitis C	○	○	○
Alcoholic fatty liver disease	○	○	○
Non-alcoholic fatty liver disease	○	○	○
Irritable bowel syndrome	○	○	○

	Never	Past	Current
Ulcerative colitis	◯	◯	◯
Crohn's disease	◯	◯	◯
Stomach or duodenal ulcer	◯	◯	◯

Neurological & Musculo-skeletal

	Never	Past	Current
Seizure disorder or epilepsy	◯	◯	◯
Migraine headaches	◯	◯	◯
Cluster headaches	◯	◯	◯
Tension headaches	◯	◯	◯
Cerebral palsy	◯	◯	◯
Multiple sclerosis	◯	◯	◯
Fibromyalgia	◯	◯	◯
Spinal cord injury	◯	◯	◯
Chronic low back pain	◯	◯	◯
Chronic musculoskeletal (joint) injury	◯	◯	◯
Rheumatoid arthritis	◯	◯	◯
Osteoarthritis	◯	◯	◯

Endocrine

	Never	Past	Current
Type 1 Diabetes mellitus	◯	◯	◯
Type 2 Diabetes mellitus	◯	◯	◯
Hypothyroidism	◯	◯	◯
Hyperthyroidism	◯	◯	◯
Congenital adrenal hyperplasia	◯	◯	◯
Cushing's disease	◯	◯	◯
Addison's disease	◯	◯	◯

Genitourinary

	Never	Past	Current
Chronic Urinary Tract Infections	◯	◯	◯
Chronic Kidney Disease	◯	◯	◯
Polycystic Kidney Disease	◯	◯	◯
Bacterial Vaginosis	◯	◯	◯
Polycystic Ovarian Syndrome	◯	◯	◯
Uterine Fibroid(s)	◯	◯	◯
Ovarian Cysts	◯	◯	◯
Benign Prostatic Hyperplasia	◯	◯	◯
Sexually Transmitted Infection (chronic)	◯	◯	◯

Sexually Transmitted Infection (acute)	○	○	○

Cancer

	Never	Past	Current
Breast	○	○	○
Non-melanoma skin cancer	○	○	○
Skin cancer	○	○	○
Lung	○	○	○
Prostate	○	○	○
Colorectal	○	○	○
Kidney	○	○	○
Bladder	○	○	○
Non-Hodgkin's lymphoma	○	○	○
Other	○	○	○

If other, please specify:

____

Psychiatric

	Never	Past	Current
Major Depressive Disorder	○	○	○
Seasonal Affective Disorder	○	○	○
Generalized Anxiety Disorder	○	○	○
Bipolar I Disorder	○	○	○
Bipolar II Disorder	○	○	○
Schizophrenia	○	○	○
Anorexia Nervosa	○	○	○
Bulemia Nervosa	○	○	○
Binge Eating Disorder	○	○	○
Eating Disorder Not Otherwise Specified (EDNOS)	○	○	○
Chronic Fatigue Syndrome	○	○	○
Insomia	○	○	○

Other diagnoses

List other diagnoses, disorders or conditions:

**Do you currently take, or have taken in the past, any of the following medications for one month or longer?**	
Thyroid hormone?	◯ Yes<br>◯ No
If yes, when did you start taking this medication (month/year)?	____________________<br>(mm/yyyy)
Do you currently take this or did you take this in the past?	◯ Current<br>◯ Past
When did you stop taking this medication? (month/year)	____________________<br>(mm/yyyy)
Dosage (in micrograms)	____________________<br>(mcg)
Prescription opioids?	◯ Yes<br>◯ No
If yes, when did you start taking this medication? (month/year)	____________________<br>(mm/yyyy)
Do you currently take this or did you take this in the past?	◯ Current<br>◯ Past
When did you stop taking this medication? (month/year)	____________________<br>(mm/yyyy)
Dosage (mg)	____________________<br>(mg)
Oral contraceptives?	◯ Yes<br>◯ No
If yes, when did you start? (month/year)	____________________<br>(mm/yyyy)
Do you currently take this or did you take this in the past?	◯ Current<br>◯ Past
When did you stop? (month/year)	____________________<br>(mm/yyyy)
Dosage (tablets)	____________________

Oral corticosteroids?

◯ Yes
◯ No

If yes, when did you start? (month/year)

____
(mm/yyyy)

Do you currently take this or did you take this in the past?

◯ Current
◯ Past

When did you stop? (month/year)

____
(mm/yyyy)

Dosage (tablets)

____

Surgeries

Have you had any of the following surgeries? (Please select all that apply)

- ☐ Appendectomy
- ☐ Breast Surgery
- ☐ Cosmetic Surgery
- ☐ Cholecystectomy
- ☐ Hysterectomy
- ☐ Joint Injury-Related Surgery
- ☐ Brain Surgery
- ☐ Colon Surgery
- ☐ Small Intestine Surgery
- ☐ Tonsillectomy
- ☐ Tubal Ligation
- ☐ Valve Replacement
- ☐ Vasectomy
- ☐ Other
- ☐ None

If 'Other,' please list:

Contraception Use

Do you currently use a form of birth control?

◯ Yes
◯ No

If yes, what form do you use? (check all that apply)

- ☐ Copper IUD (Paragard)
- ☐ Hormonal IUD (Mirena, Kyleena, Liletta, and Skyla)
- ☐ Contraceptive Implant
- ☐ Birth Control Shot
- ☐ Birth Control Pill
- ☐ Birth Control Patch
- ☐ Vaginal Ring
- ☐ Diaphragm and Cervical Cap
- ☐ Male Condom
- ☐ Female Condom
- ☐ Withdrawal
- ☐ Sponge
- ☐ Natural Family Planning and Fertility Awareness
- ☐ Lactational Amenorrhea Method (LAM)
- ☐ Spermicide
- ☐ Abstinence
- ☐ Emergency Contraception (Morning After Pill)

Healthcare Utilization

Please answer the following questions based on your utilization of healthcare in the past 12 months.

	Never	1 time in the last year	2-4 times in the last year	5-11 times in the last year	1 time per month	More than 1 time per month
1. In the past twelve months, how often have you been seen by a conventional medical provider? This includes MD, DO, NP, or PA providers.	○	○	○	○	○	○
In the past twelve months, how often have your been seen by a naturopathic physician?	○	○	○	○	○	○
In the past twelve months, how often have your been seen by an acupuncturist (excluding TCM or CCM herbal medicine)?	○	○	○	○	○	○
In the past twelve months, how often have your been seen by a Chinese medicine provider (including TCM or CCM herbal medicine)?	○	○	○	○	○	○
In the past twelve months, how often have your been seen by a craniosacral therapist?	○	○	○	○	○	○

In the past twelve months, how often have your been seen by a massage therapist?	○	○	○	○	○	○
In the past twelve months, how often have your been seen by a chiropractor?	○	○	○	○	○	○
In the past twelve months, how often have your been seen by a yoga therapist?	○	○	○	○	○	○
In the past twelve months, how often have your been seen by a mental health provider? This includes counselors, psychologists and psychiatrists.	○	○	○	○	○	○

List Of Medications And Supplements Repeating

Please complete the survey below.

Thank you!

Medication Use

Please list any prescription pharmaceuticals or over-the-counter medications you are currently taking or have taken in the past month. Do NOT include prescribed vitamins, herbs or other supplements.

1) Are you still taking [previous-event-name][med_name_v3r]?
   - ◯ Yes
   - ◯ No
   - ◯ N/A - This is a new medication

2) Medication 1 Name ______________________

3) Dose per unit (e.g., per capsule, tablet, drop, spoonful, pellet) ______________________
   (Provide numeric value only)

4) Select dosage unit:
   - ◯ micrograms
   - ◯ milligrams
   - ◯ grams
   - ◯ milliliters
   - ◯ liters
   - ◯ IU
   - ◯ Other

5) Dosage per day ______________________

6) Number of days per month ______________________

7) Start Date: mm/yyyy ______________________

8) Photo (front) of product label

9) Photo (back) of product label

10) Reason for use (check one of more):

- ☐ Stress
- ☐ Sleep
- ☐ Digestive
- ☐ Hormonal
- ☐ Cognition
- ☐ Musculoskeletal
- ☐ Anti-Inflammatory
- ☐ Immune
- ☐ Mood
- ☐ Respiratory
- ☐ Cardiac
- ☐ Skin
- ☐ Energy
- ☐ Weight Management
- ☐ General Health
- ☐ Other

11) If other, please specify: ______________________

12) Number of Medications ______________________

List Of Supplements Repeating

Please complete the survey below.

Thank you!

Supplement History

Please list any herbal supplements, vitamins, minerals, or other nutritional supplements you are currently taking or have taken in the past month. This list should include any tinctures, vitamins, minerals, herbs, Chinese formulas, or homeopathic preparations.

1) Are you still taking [previous-event-name][sup_name_v3r] supplement?
   - ◯ Yes
   - ◯ No
   - ◯ N/A - This is a new supplement

2) Supplement 1 Name ______________________________

3) Manufacturer Name ______________________________

4) Dose per unit (e.g., per capsule, tablet, drop, spoonful, pellet) ______________________________
   (Provide numeric value only)

5) Select dosage unit:
   - ◯ micrograms
   - ◯ milligrams
   - ◯ grams
   - ◯ milliliters
   - ◯ liters
   - ◯ IU
   - ◯ Other

6) Dosage per day ______________________________

7) Number of days per month ______________________________

8) Start Date: mm/yyyy ______________________________

9) Photo (front) of product label

10) Photo (back) of product label

11) Reason for use (check one of more):

- ☐ Stress
- ☐ Sleep
- ☐ Digestive
- ☐ Hormonal
- ☐ Cognition
- ☐ Musculoskeletal
- ☐ Anti-Inflammatory
- ☐ Immune
- ☐ Mood
- ☐ Respiratory
- ☐ Cardiac
- ☐ Skin
- ☐ Energy
- ☐ Weight Management
- ☐ General Health
- ☐ Other

12) If other, please specify: ______________________

Substance Use Survey

Please complete the survey below.

All information obtained through this questionnaire will be kept confidential. Your responses will become part of your research for this study but will not become part of your medical records at NUNM.

Thank you!

All information obtained through this questionnaire will be kept confidential. Your responses will become part of your research record for this study but will not become part of your medical records at NUNM.

These questions are about substance use in the past month. For each question, please mark only one answer.

Tobacco: frequency

		Never	1 time in the last month	2-3 times in the last month	1 time per week	2 times per week	3-4 times per week	5-6 times per week	1 time per day	2 or more times per day
1)	1. In the past month, how often have you used tobacco? This includes cigarettes, cigars, pipes, and smokeless tobacco.	○	○	○	○	○	○	○	○	○

e-cigarettes or vaping: frequency

		Never	1 time in the last month	2-3 times in the last month	1 time per week	2 times per week	3-4 times per week	5-6 times per week	1 time per day	2 or more times per day
2)	2. In the past month, how often have you used nicotine-based e-cigarettes or vaping?	○	○	○	○	○	○	○	○	○

Cannabis: frequency

		Never	1 time in the last month	2-3 times in the last month	1 time per week	2 times per week	3-4 times per week	5-6 times per week	1 time per day	2 or more times per day
3)	3. In the past month, how often have you smoked or "vaped" Cannabis?	○	○	○	○	○	○	○	○	○

Edible Cannabis: frequency

		Never	1 time in the last month	2-3 times in the last month	1 time per week	2 times per week	3-4 times per week	5-6 times per week	1 time per day	2 or more times per day
4)	4. In the past month, how often have you used edible Cannabis?	○	○	○	○	○	○	○	○	○

Recreational Drugs: frequency

		Never	1 time in the last month	2-3 times in the last month	1 time per week	2 times per week	3-4 times per week	5-6 times per week	1 time per day	2 or more times per day
5)	5. In the past month, how often have you used recreational drugs? This includes cocaine, ecstasy, methamphetamine, heroin, and any other illicit drugs.	○	○	○	○	○	○	○	○	○

Alcohol: frequency

		Never	1 time in the last month	2-3 times in the last month	1 time per week	2 times per week	3-4 times per week	5-6 times per week	1 time per day	2 or more times per day
6)	6. In the past month, how often have you used alcohol? This includes beer, wine, cider, and hard liquor.	○	○	○	○	○	○	○	○	○

Stress Management and Self-Care Survey

Please complete the survey below.

All information obtained through this questionnaire will be kept confidential. Your responses will become part of your research record for this study but will not become part of your medical records at NUNM.

Thank you!

All information obtained through this questionnaire will be kept confidential. Your responses will become part of your research record for this study but will not become part of your medical records at NUNM.

These questions are about stress management and self-care activities you have done in the past month. When answering, please include all times you have participated in these activities over the past month, even if it was only for a short time. For each question, please mark only one answer.

Mindfulness Meditation: frequency

	Never	1 time in the last month	2-3 times in the last month	1 time per week	2-3 times per week	More than 3 times per week
1. In the past month, how often have you participated in mindfulness meditation? This includes Vipassana, Zen Buddhist meditation, Mindfulness-Based Stress Reduction, and Mindfulness-Based Cognitive Therapy.	○	○	○	○	○	○

Prayer: frequency

	Never	1 time in the last month	2-3 times in the last month	1 time per week	2-3 times per week	More than 3 times per week
2. In the past month, how often have you used prayer?	○	○	○	○	○	○

Asana yoga: frequency

	Never	1 time in the last month	2-3 times in the last month	1 time per week	2-3 times per week	More than 3 times per week
3. In the past month, how often have you participated in an asana yoga practice?	○	○	○	○	○	○

Pranayama yoga practice: frequency

	Never	1 time in the last month	2-3 times in the last month	1 time per week	2-3 times per week	More than 3 times per week
4. In the past month, how often have you participated in a pranayama yoga practice?	○	○	○	○	○	○

Tai Chi or Qigong: frequency

	Never	1 time in the last month	2-3 times in the last month	1 time per week	2-3 times per week	More than 3 times per week
5. In the past month, how often have you participated in, or practiced, Tai Chi or Qigong?	○	○	○	○	○	○

Other self-care activities

	No	Yes
6. Do you do other self-care activities to manage your stress?	○	○

7. What other self-care activity(ies) or practices do you do to help manage your stress?

____

	Never	1 time in the last month	2-3 times in the last month	1 time per week	2-3 times per week	More than 3 times per week
8. How often do you do the activity(ies) from above?	○	○	○	○	○	○
