# Supplementary material for: Meditative practices, stress and sleep among students studying complementary and integrative health: a cross-sectional analysis
Source: BMC Complement Med Ther. 2022 May 5;22:127. doi: 10.1186/s12906-022-03582-5 (PMC9070612; doi:10.1186/s12906-022-03582-5)
Supplement: Supplementary file 2 — Additional file 2. [file 12906_2022_3582_MOESM2_ESM.pdf]

Perceived Stress Scale (PSS-10)

Please complete the survey below.

All information obtained through this questionnaire will be kept confidential. Your responses will become part of your research record for this study but will not become part of your medical records at NUNM.

Thank you!

The Perceived Stress Scale (PSS) is a classic stress assessment instrument. The tool, while originally developed in 1983, remains a popular choice for helping us understand how different situations affect our feelings and our perceived stress. The questions in this scale ask about your feelings and thoughts during the last month. In each case, you will be asked to indicate how often you felt or thought a certain way. Although some of the questions are similar, there are differences between them and you should treat each one as a separate question. The best approach is to answer fairly quickly. That is, don't try to count up the number of times you felt a particular way; rather indicate the alternative that seems like a reasonable estimate.

For each question choose from the following alternatives:
0 = Never / 1 = Almost Never / 2 = Sometimes / 3 = Fairly Often / 4 = Very Often

1) 1. In the last month, how often have you been upset because of something that happened unexpectedly?

◯ 0 = Never
◯ 1 = Almost Never
◯ 2 = Sometimes
◯ 3 = Fairly Often
◯ 4 = Very Often

2) 2. In the last month, how often have you felt that you were unable to control the important things in your life?

◯ 0 = Never
◯ 1 = Almost Never
◯ 2 = Sometimes
◯ 3 = Fairly Often
◯ 4 = Very Often

3) 3. In the last month, how often have you felt nervous and stressed?

◯ 0 = Never
◯ 1 = Almost Never
◯ 2 = Sometimes
◯ 3 = Fairly Often
◯ 4 = Very Often

4) 4. In the last month, how often have you felt confident about your ability to handle your personal problems?

◯ 0 = Never
◯ 1 = Almost Never
◯ 2 = Sometimes
◯ 3 = Fairly Often
◯ 4 = Very Often

5) 5. In the last month, how often have you felt that things were going your way?

◯ 0 = Never
◯ 1 = Almost Never
◯ 2 = Sometimes
◯ 3 = Fairly Often
◯ 4 = Very Often

6) 6. In the last month, how often have you found that you could not cope with all the things that you had to do?

◯ 0 = Never
◯ 1 = Almost Never
◯ 2 = Sometimes
◯ 3 = Fairly Often
◯ 4 = Very Often

7) 7. In the last month, how often have you been able to control irritations in your life?

- ◯ 0 = Never
- ◯ 1 = Almost Never
- ◯ 2 = Sometimes
- ◯ 3 = Fairly Often
- ◯ 4 = Very Often

8) 8. In the last month, how often have you felt that you were on top of things?

- ◯ 0 = Never
- ◯ 1 = Almost Never
- ◯ 2 = Sometimes
- ◯ 3 = Fairly Often
- ◯ 4 = Very Often

9) 9. In the last month, how often have you been angered because of things that happened that were outside of your control?

- ◯ 0 = Never
- ◯ 1 = Almost Never
- ◯ 2 = Sometimes
- ◯ 3 = Fairly Often
- ◯ 4 = Very Often

10) 10. In the last month, how often have you felt difficulties were piling up so high that you could not overcome them?

- ◯ 0 = Never
- ◯ 1 = Almost Never
- ◯ 2 = Sometimes
- ◯ 3 = Fairly Often
- ◯ 4 = Very Often

11) PSS Total Score: ______________________

Cohen, S., Kamarck, T, and Mermelstein, R. "A Global Measure of Perceived Stress." Journal of Healthand Social

Behavior, Vo. 24, No. 4 (Dec.,1983), Appendix A.
