# Supplementary material for: Meditative practices, stress and sleep among students studying complementary and integrative health: a cross-sectional analysis
Source: BMC Complement Med Ther. 2022 May 5;22:127. doi: 10.1186/s12906-022-03582-5 (PMC9070612; doi:10.1186/s12906-022-03582-5)
Supplement: Supplementary file 3 — Additional file 3. [file 12906_2022_3582_MOESM3_ESM.pdf]

PROMIS - 29 Profile v2.0

Please complete the survey below.

All information obtained through this questionnaire will be kept confidential. Your responses will become part of your research for this study but will not become part of your medical records at NUNM.

Thank you!

Please respond to each item by choosing one answer per statement.

Physical Function

		Without any difficulty	With a little difficulty	With some difficulty	With much difficulty	Unable to do
1)	1. Are you able to do chores such as vacuuming or yard work?....	○	○	○	○	○
2)	2. Are you able to go up and down stairs at a normal pace?....	○	○	○	○	○
3)	3. Are you able to go for a walk of at least 15 minutes?....	○	○	○	○	○
4)	4. Are you able to run errands and shop?....	○	○	○	○	○

Anxiety

In the past 7 days...

		Never	Rarely	Sometimes	Often	Always
5)	5. I felt fearful....	○	○	○	○	○
6)	6. I found it hard to focus on anything other than my anxiety....	○	○	○	○	○
7)	7. My worries overwhelmed me....	○	○	○	○	○
8)	8. I felt uneasy....	○	○	○	○	○

Depression

In the past 7 days...

		Never	Rarely	Sometimes	Often	Always
9)	9. I felt worthless....	○	○	○	○	○
10)	10. I felt helpless....	○	○	○	○	○
11)	11. I felt depressed....	○	○	○	○	○
12)	12. I felt hopeless....	○	○	○	○	○

Fatigue

During the past 7 days...

		Not at all	A little bit	Somewhat	Quite a bit	Very much
13)	13. I feel fatigued....	◯	◯	◯	◯	◯
14)	14. I have trouble starting things because I am tired....	◯	◯	◯	◯	◯
15)	15. How run-down did you feel on average?...	◯	◯	◯	◯	◯
16)	16. How fatigued were you on average?....	◯	◯	◯	◯	◯

Sleep Disturbance

In the past 7 days...

		Very poor	Poor	Fair	Good	Very good
17)	17. My sleep quality was....	◯	◯	◯	◯	◯

In the past 7 days...

		Not at all	A little bit	Somewhat	Quite a bit	Very much
18)	18. My sleep was refreshing....	◯	◯	◯	◯	◯
19)	19. I had a problem with my sleep....	◯	◯	◯	◯	◯
20)	20. I had difficulty falling asleep	◯	◯	◯	◯	◯

Ability to Participate in Social Roles and Activities

		Never	Rarely	Sometimes	Usually	Always
21)	21. I have trouble doing all of my regular leisure activities with others....	◯	◯	◯	◯	◯
22)	22. I have trouble doing all of the family activities that I want to do....	◯	◯	◯	◯	◯
23)	23. I have trouble doing all of my usual work (include work at home)....	◯	◯	◯	◯	◯
24)	24. I have trouble doing all of the activities with friends that I want to do....	◯	◯	◯	◯	◯

**Pain Interference** **In the past 7 days...**	Not at all	A little bit	Somewhat	Quite a bit	Very much
25) 25. How much did pain interfere with your day to day activities?....	◯	◯	◯	◯	◯
26) 26. How much did pain interfere with work around the home?....	◯	◯	◯	◯	◯
27) 27. How much did pain interfere with your ability to participate in social activities?....	◯	◯	◯	◯	◯
28) 28. How much did pain interfere with your household chores?....	◯	◯	◯	◯	◯

**Pain Intensity** **In the past 7 days...**	No Pain	1	2	3	4	5	6	7	8	9	Worst imagin able pain
29) 29. How would you rate your pain on average?....	◯	◯	◯	◯	◯	◯	◯	◯	◯	◯	◯

© 2008-2013 PROMIS Health Organization and PROMIS Cooperative Group
