# Supplementary material for: Meditative practices, stress and sleep among students studying complementary and integrative health: a cross-sectional analysis
Source: BMC Complement Med Ther. 2022 May 5;22:127. doi: 10.1186/s12906-022-03582-5 (PMC9070612; doi:10.1186/s12906-022-03582-5)
Supplement: Supplementary file 4 — Additional file 4. [file 12906_2022_3582_MOESM4_ESM.pdf]

Table 2: Baseline Characteristics of cohort participants enrolled prior to the COVID-19 and during the COVID-19 pandemic

Characteristic	**Pre-COVID** (n = 89)	**COVID** (n = 9)	**P-value**
Age, mean (SD)	28.8 (5.75)	34.1 (8.05)	0.01
Female Sex, number (%)	74 (83.5%)	8 (88.8%)	0.68
White, Non-Hispanic, n(%)	66 (74.2%)	7 (77.8%)	0.81
BMI, mean (SD)	23.8 (3.95)	24.3 (5.7)	0.70
Current Smoker, n(%)	12 (12.4%)	1 (11.1%)	0.91
Alcohol drinks per week, mean (SD)	1.39 (2.01)	0.89 (1.29)	0.47
Physical Activity Level, n (%)			0.61
Sedentary	8 (9.2%)	1 (11.1%)	
Low Activity	25 (27.6%)	2 (22.2%)	
Active	42 (47.1%)	3 (33.3%)	
Very Active	14 (16.1 %)	3 (33.3%)	
AUC Cortisol (ng/mL), median (IQR)			
Morning	6.3 (4.4 – 8.0)	4.6 (4.2 – 5.1)	0.15
Noon	2.0 (1.5 – 2.8)	1.9 (1.5 – 2.3)	0.36
Evening	1.2 (0.9 – 1.7)	1.0 (0.9 – 1.7)	0.58
Night	0.7 (0.5 – 1.0)	0.6 (0.5 – 1.0)	0.90
PSS-10 score, mean (SD)	15.6 (6.0)	12.1 (7.2)	0.106
PROMIS-Sleep, mean (SD)	48.2 (7.4)	49.0 (8.3)	0.751
Meditative Practice, n(%)			0.11
Never	15(17.0%)	2 (22.2%)	
1x/month	6 (6.8%)	3 (33.3%)	
2-3x/month	11 (12.5%)	0	
1x/week	14 (16%)	1 (11.1%)	
2-3x/week	13 (15%)	0	
>3x/week	29 (33%)	3 (33.3%)	
Mind Body Practices, n(%)			
Prayer ≥ 1 per week, n(%)	30(33.7%)	2 (22.2%)	0.73
Asana Yoga ≥ 1 per week, n(%)	23 (25.8%)	5 (55.5%)	0.11
Pranayama Yoga ≥ 1 per week, n(%)	16 (18.0%)	1 (11.1%)	0.70
Tai-Chi ≥ 1 per week, n(%)	12 (13.6%)	6 (66.7%)	0.002
Other ≥ 1 per week, n(%)	0	0	-

IQR (Interquartile range) BMI (Body Mass Index) AUC (Area Under the Curve) PSS-10 (10-item Perceived Stress Scale) PROMIS-Sleep (Patient Reported Outcome Measures Information System-Sleep Score) ng/mL (nanogram per milliliter)
